# From Toxins Targeting Ligand Gated Ion Channels to Therapeutic Molecules

**DOI:** 10.3390/toxins3030260

**Published:** 2011-03-21

**Authors:** Adak Nasiripourdori, Valérie Taly, Thomas Grutter, Antoine Taly

**Affiliations:** 1 Department of Biology, Faculty of Sciences, Arak University, Iran; Email: nasiripour_a@yahoo.com; 2 Laboratory of Chemical Biology, Institut de Science et d'Ingénierie Supramoléculaires; ISIS/Université de Strasbourg, CNRS-UMR 7006, 8, allée Gaspard Monge, BP 70028, F-67083, Strasbourg Cedex, France; Email: vtaly@unistra.fr; 3 Laboratoire de Biophysicochimie des Récepteurs Canaux, UMR 7199 “Conception et Application de Molécules Bioactives” CNRS-Université de Strasbourg, 74 Route du Rhin-BP 60024, 67401 Illkirch Cedex, France; Email: grutter@bioorga.u-strasbg.fr

**Keywords:** nAChR, P2X, GABA, Glycine, Serotonin, NMDA, AMPA, Kainate

## Abstract

Ligand-gated ion channels (LGIC) play a central role in inter-cellular communication. This key function has two consequences: (i) these receptor channels are major targets for drug discovery because of their potential involvement in numerous human brain diseases; (ii) they are often found to be the target of plant and animal toxins. Together this makes toxin/receptor interactions important to drug discovery projects. Therefore, toxins acting on LGIC are presented and their current/potential therapeutic uses highlighted.

## 1. Introduction and Scope of the Review

### 1.1. To be Poisonous or Not: The Dose Effect

When thinking of toxic compounds, researchers often have in mind the famous citation of Paracelsus (Philippus Theophrastus Aureolus Bombastus von Hohenheim): "The dose makes the poison" or in its more complete version "All things are poison and nothing is without poison. Solely the dose determines that a thing is not a poison." [1]. Although this 16th century concept is now debated, Paracelsus is still recognized as one of the fathers of "Toxicology" as it is envisioned today.

Medical drugs and toxins are good illustrations of this adage. On the one hand, medical drugs are often perceived as non-poisonous because of their potential benefit for humans. However, it is now well known that widely used drugs like Paracetamol℘ or Ibuprofen℘ can be highly toxic at relatively moderate doses [2]. On the other hand, toxins are generally perceived as dangerous for humans because of their possible accumulation in the alimentary chain or their use as biological weapons (e.g., anatoxin-a). However, following Paracelsus, lowering the dose might allow to make them non-toxic. Solving the issue of toxicity would pave the way to turn them into medicine. Indeed, in addition to their use as an invaluable source of ligands for studying structural or functional properties of their molecular targets [3], these molecules are now increasingly interesting to researchers for their use as medicine or cosmetic products [4].

### 1.2. Toxins as Biological Poisons

The word 'toxin' was first introduced by Ludwig Brieger as a name for poisons made by infectious agents [5]. These biological poisons allow the organisms to survive difficult environmental situations where the toxins are advantageous for prey capture or defense [6]. Plant toxins (e.g., nicotine) often function as protection against certain animals. In animals, toxins have similar defense potential and are also used to capture prey. Toxicity might, however, be less directly connected with environmental situations as in the case of fish and shellfish that become poisonous after feeding on toxic plants or algae.

Toxins are nowadays usually defined as poisonous substances produced by living organisms including bacteria, microalgae, plants or fungi. We will use here this definition and therefore restrict ourselves to natural substances affecting an animal.

### 1.3. Scope of the Review

The knowledge of toxins acting on the ligand gated ion channels (LGIC) is dispersed and not homogeneous. Some toxins were isolated and chemically characterized, but poorly studied on the LGIC afterwards. Others found very wide applications and are frequently used in research or even as therapeutic intervention. This in-homogeneity could be due to the fact that the study of toxins acting on LGIC is the intersection of two relatively separated fields: toxinology on one hand and the study of the LGIC on the other hand. Another explanation is that many of the toxins described below were discovered and characterized (and then forgotten?), before the diverse LGIC had been identified. We therefore decided to construct a list of toxins targeting the LGIC that would be as exhaustive as possible.

This compilation of toxins targeting the LGIC should be useful as toxins constitute a relatively unbiased—in term of chemical space covered—source of ligand structures that can be used: (i) as a source of inspiration for drug design, as much as hits identified from high-throughput screening experiments; (ii) in structure/activity relationship studies; (iii) in virtual screening studies. The structure of a representative member of each family of the ligand-gated ion channels has been very recently solved by X-ray crystallography making these studies timely. 

## 2. Toxins Targeting the Ligand Gated Ion Channels

LGIC are allosteric proteins (for a recent review on the allosteric nature of nicotinic receptors, see [7]). Indeed, their functioning implies that they are in equilibrium between a few states, switching from the resting state to an active state with an open ion-channel, and eventually a desensitized state. In this framework, the ligands (agonists, antagonists and allosteric modulators) act by altering the equilibrium, *i.e.*, they stabilize the state for which they have the highest affinity. The endogenous agonist binds, by definition, in the orthosteric binding site. The other ligands, notably toxins, bind either at the same site (agonists and competitive antagonists), or in other binding sites (allosteric modulators and non-competitive antagonists). 

LGIC are oligomeric receptors made by the association of identical or homologous subunits [8]. The LGIC superfamilly can be subdivided into three families depending on the number of monomers composing an oligomer: the pentameric, tetrameric and trimeric LGIC.

**Figure 1:**
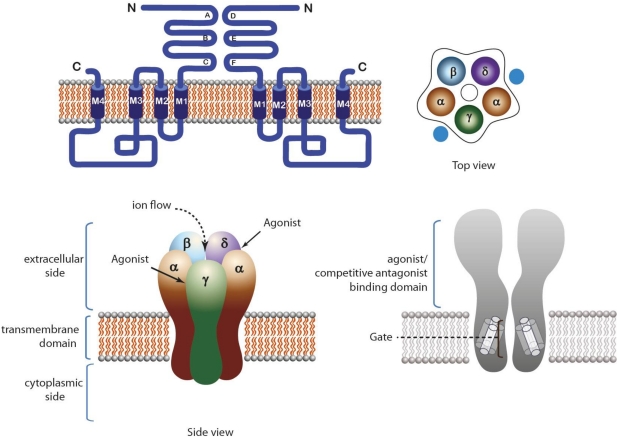
Structure of pentameric ligand-gated ion channels (LGIC). Muscle type nAChR is taken as an example. Top left: Topology of the receptor. Top right: Top view of the receptor. Bottom left: Side view of the receptor showing the extracellular, intracellular and transmembrane domains. Agonist binding sites are located at subunit interfaces in the extracellular side of the receptor. Bottom right: Longitudinal cross section of the receptor, showing the pore domain.

The pentameric family encompasses the nicotinic acetylcholine receptors (nAChRs; α1-10, β1-4, γ, δ, ε), the Gamma-aminobutyric acid (GABA) receptors (α1-6, β1-3, γ1-3, δ, ε, θ, π, ρ1-3) receptors, the Serotonin (5-hydroxytryptamine, 5HT3) receptors (5-HT3A-E) and the Glycine receptors (α1-3, β) [9,10]. The endogenous agonist binding site lies at the interface between subunits (Figure 1). The location of alternative binding sites has been identified at homologous interfaces (e.g., benzodizepines for the GABA receptors) and in the transmembrane domain [11]. 

**Figure 2:**
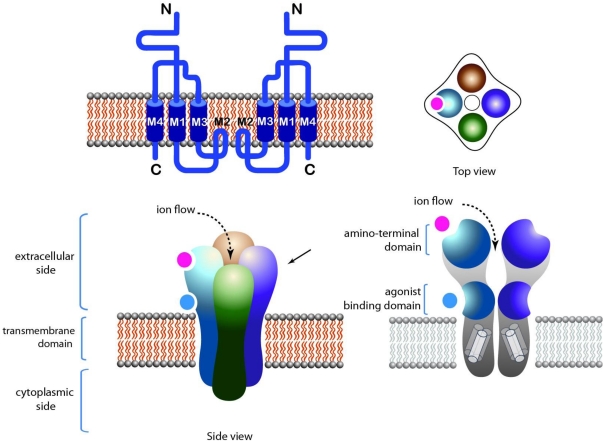
Structure of tetrameric LGIC. NMDA receptor is taken as an example. Top left: Topology of the receptor. Top right: Top view of the receptor. Bottom left: Side view of the receptor showing the extracellular, intracellular and transmembrane domains. Agonist binding sites are located in juxta-membrane domains in the extracellular side of the receptor. Bottom right: Longitudinal cross section of the receptor, showing the pore domain.

The tetrameric family of LGIC consists of glutamate receptors. This LGIC family contains the α-amino-3-hydroxyl-5-methyl-4-isoxazole-propionate (AMPA) receptors (GluA1-4), kainate receptors (GluK1-5) and N-methyl D-aspartate (NMDA) receptors (GluN1, GluN2A-D, GluN3A-B). The agonist binding site of tetrameric LGIC lies inside monomers (Figure 2). The agonists and alternative binding sites are known from biochemical and structural studies [12].

**Figure 3:**
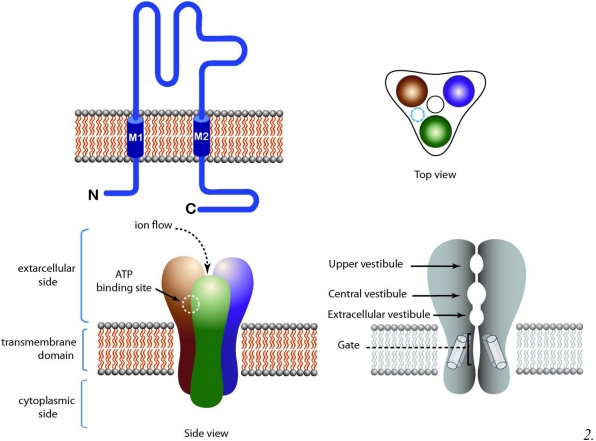
A schematic view of trimeric LGIC (P2X receptor). Top left: Topology of the receptor. Top right: Top view of the receptor. Bottom left: Side view of the receptor showing the extracellular, intracellular and transmembrane domains. Agonist binding sites are presumably located at subunit interfaces in the extracellular side of the receptor. Bottom right: Longitudinal cross section of the receptor, showing the pore domain.

The trimeric family of LGIC is made by P2X receptors (P2X1-7). The knowledge of the binding site is much more restricted compared to that of pentameric and tetrameric LGIC: the binding site for ATP has been tentatively localized in a cavity at the interface between subunits (Figure 3).

Below are listed selected toxins, known to act on the various LGIC, that are important for historical or pharmacological reasons. This series of toxins is complemented by a list presented as an appendix (Section 5).

### 2.1. Nicotinic Acetylcholine Receptors

Nicotinic receptors are arguably the most well-known LGIC to date. This is probably due to their very early discovery and the large number of toxins blocking them. Historically, the pharmaceutical knowledge of the cholinergic system has emerged with the “discovery” of **curares** by Spanish explorers in South America during the 16th century. Indeed, curares were used there by local tribes for hunting [1]. It was found during the 19th century that curares block the synaptic transmission at the level of the neuromuscular junction [13] therefore paralyzing the prey. Similar usage of curares have also been reported in Africa [14], and Malaysia [15]. Toxins had an invaluable contribution to the emergence of the notion of LGIC as nAChR where first defined as the “nicotine and curare receptive substance” [16].

**Figure 4:**
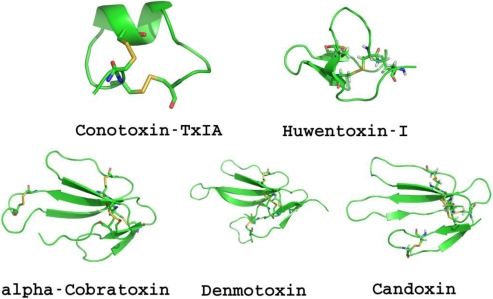
Representative protein toxins targeting the nicotinic acetylcholine receptors.

Another historically significant toxin targeting the nAChR is **α-Bungarotoxin** (reviewed in [17]). It was first used to isolate the nAChR [18,19]. The venoms of marine cone snails represent a rich combinatorial-like library of evolutionarily selected, neuropharmacologically active peptides called **conotoxins** (Figure 4, Table 1) that target a wide variety of receptors and ion-channels [20]. The subtype specific snake α-neurotoxins and cone snail α-conotoxins are still widely used to probe receptor structure and function in native tissues and recombinant systems [21].

**Figure 5:**
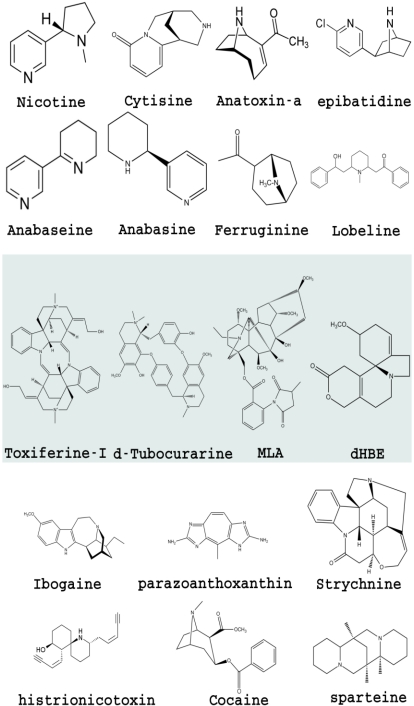
Representative alkaloids targeting the nicotinic acetylcholine receptors. Top: agonists, middle: competitive antagonists, bottom; non-competitive antagonists.

**Nicotine** is a highly toxic alkaloid proposed to serve as an insecticide protecting Tobacco plants [22]. It is the prototypical agonist at nicotinic cholinergic receptors (Figure 5) in comparison to muscarinic receptors [23]. Tobacco extract was used as an insecticide for centuries [24], perhaps as early as 1690 [25]. The effect relies on the presence of nicotine and anabasine (see Section 5). Moreover, nicotine and anabasine were still in use in the early 20th century (see below). Nicotine is also important medically because it is thought to be responsible for tobacco addiction through the stimulation of α4β2 nAChR on dopaminergic neurons of the ventral tegmental area (VTA) [26].

**Table 1:** α-conotoxins acting on nAChRs.

Name	*Conus* specie	Target	References
**GI, GIA, GII**	*geographus*	Muscle nAChR	[27]
**MI**	*magus*	Muscle nAChR	[28]
**SI, SIA and SII**	*striatus*	Selectivity for the distinct interfaces (α/γ or α/δ) of the muscle-type nAChR	[28,29]
**ImI, ImII**	*imperialis*	Selective for α7 nAChR but also effective on α3β4, α3β2	[30]
**BuIA**	* bullatus*	Highest potency for α3- and α6−containing nAChRs	[31]
**CnIA, CnIB **	*consors*	Muscle nAChR	[32]
**Ac1.1a, Ac1.1b**	*achatinus*	Muscle nAChR	[33]
**EI**	*ermineus*	Selective for muscle nAChR, also effective on α3β4, α4β2	[34]
**PnIB, (A10L)-PnIA**	*pennaceus*	Selective for α7, α3β4, α3β2 nAChR	[35,36]
**GIC**	*geographus*	Selective for α3β2	[37]
**MII **	*magnus*	Selective for α3β2, α3β2β3, α6* nAChR	[38,39]
**PIA **	*purpurascens*	Selective for α6β2, α6β4, α6α3β2(β3), α6α3β4	[40]
**PIB**	*purpurascens*	Muscle nAChR	[41]
**GID**	*geographus*	α7, α3β2, α4β2	[42,43]
**AuIA, AuIB and AuIC **	*aulicus*	Selectively blocks α3β4 nAChRs	[44]
**EPI **	*episcopatus*	Selective for α7, α3β2, α3β4	[45,46]
**AnIB**	*anemone*	α7, α3β2	[47]
**Vc1.1**	*victoriae*	α9, α3β4, α3(α5)β2	[48,49]
**ArIA, ArIB**	*arenatus*	α7, α3β2α6α3β2β3	[50]
**PeIA**	*pergrandis*	α9α10,α6α3β2β3,α3β2	[51]
**OmIA**	*omaria*	α7, α3β2	[52]
**TxIA**	*textile*	α3β2	[53]
**Lp1.1**	*leopardus*	α3β2,α6α3β2	[54]
**SrIA, SrIB**	*spurious*	α4β2, muscle type nAChRs	[55]

**β-Erythroidine** is isolated from the coral tree *Erythrina crista-galli.* It acts as a competitive antagonist of nicotinic receptors (it targets α4β4, α4β2 and α3β2* receptors and has weak affinity for the muscle type and α3β4) [56]. At high concentrations, they are noncompetitive blockers of possibly all nicotinic receptors subtypes [57,58,59]. **Erysodine** is a structurally related Erythrina Alkaloid acting on α4β2 and α3β2* nAChR. Both compounds are weak binders to α7 nAChR explaining that they are used to discriminate between different nAChR subtypes although they are relatively non-selective.

**Methyllycaconitine (MLA)**, is extracted from *Delphinium* species [60] and is a potent and highly selective α7 nAChR antagonist [61,62]. MLA is largely used for this property as a pharmacological tool in research [63]. MLA together with additional alkaloids in *Delphinium* species (**nudicauline, 14-deacetylnudicauline**, **barbinine** and **deltaine**) have also been found to act on nAChRs blocking the neuromuscular junction which may be related to the *Delphinium* species involvement in cattle poisoning [64,65].

### 2.2. GABA-A Receptors

The toxins of the GABA receptors are, as for the nAChR, of different categories (agonists, antagonists and allosteric modulators) as illustrated by the examples described below.

**Figure 6:**
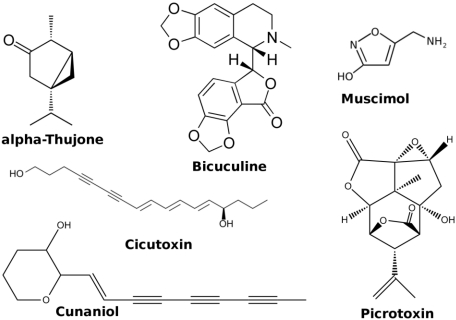
Representative toxins targeting the GABA receptors.

**α-thujone** is extracted from the wormwood *Artemisia absinthium* and is found in absinthe [66]. It is a negative allosteric modulator of GABA-A receptors (Figure 6) resulting in convulsant activity [67]. **α-thujone** also antagonizes 5HT3 receptors [68].

**Bicuculline**, isolated from *Dicentra cucullaria*, is a competitive antagonist of GABA-A receptors causing convulsions [66]. 

**Muscimol** is an agonist extracted from *Amanita muscaria* partly responsible for the toxic effect of the mushroom [66].

**Picrotoxin** is a non-competitive antagonists isolated from *Menispermaceae.* Binding modes for picrotoxin have been proposed in the ion channel [69,70,71].

### 2.3. Glycine Receptors

**Strychnine** is found in the seeds of the Strychnine tree (*Strychnos nux-vomica*). Strychnine causes muscular convulsions and eventually death through asphyxia or sheer exhaustion. It is used as a pesticide, particularly for killing small vertebrates such as birds and rodents. Strychnine participates in the pharmacological differentiation of receptors responsive to glycine. Indeed, some NMDA receptors are also activated by glycine but are strychnine insensitive [72].

### 2.4. Serotonin Receptors

The serotonin receptors have very few known toxins: **Conotoxin GVIIA** (σ-conotoxin; a large 41 amino-acids conotoxin [73]) and **d-tubocurarine**.

d-Tubocurarine is a mono-quaternary alkaloid obtained from the bark and stems of *Chondrodendron tomentosum*. d-Tubocurarine blocks nAChRs at the neuromuscular junction but also acts on serotonin receptors [74]. d-Tubocurarine is the archetypal **curare**. As neuromuscular blockers, curares can be used as skeletal muscle relaxants and were indeed introduced in anesthesia in 1942 [75]. Curares are active only by injection. They are harmless if taken orally because curare compounds are too large and too highly charged to pass through the lining of the digestive tract to be absorbed into the blood. This explains how they can be used to kill prey that will be later ingested. 

### 2.5. NMDA Receptors

**Ageltoxin (agatoxin)** are arylamine toxins (the α-agatoxins) found in the venom of the spider *Agelenopsis aperta*. They paralyze insects by blocking glutamatergic neuromuscular transmission [76]. Ageltoxins are thought to be non-competitive channel blockers specific for NMDA receptors [76,77].

**Figure 7:**
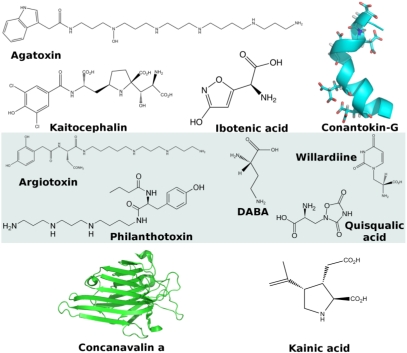
Representative toxins targeting the glutamate receptors. Top: NMDA receptors; Middle: AMPA receptors; Bottom: Kainate receptors.

**Conantokins** are found in the venom from *Conus* fish hunting snails [78]. The conantokins (G, L, R and T) form a class of peptides that inhibit competitively NMDA receptors [79,80]. Interestingly conantokins possess a large number of γ-carboxyglutamic acid residues (Figure 7) [81]. One of the γ−carboxyglutamic acid residues is thought to participate in the selectivity of conantokin G [82].

### 2.6. AMPA Receptors

**Quisqualic acid** is isolated from the seeds of *Quisqualis indica*. Quisqualic acid is an agonist at AMPA receptors [83]. Quisqualate used to be the prototypical ligand of AMPA receptors, which were therefore called quisqualate receptors. However, this name has been abandoned as quisqualate also acts at metabotropic glutamate receptors [83].

### 2.7. Kainate Receptors

**Kainic acid** was first isolated from the red alga *Digenia simplex*, where it might play a defense role, [84] and is also found in other algae [85]. Kainic acid is the prototypical agonist defining the Kainate subtype of glutamate receptors. The toxin is associated with human poisoning through the consumption of mussels that eat the algae.

### 2.8. P2X Receptors

**Purotoxin** are P2X receptors modulators which were isolated from the venom of the wolf spider Geolycosa sp [86]. Later, purotoxin has been isolated and shown to be a non-competitive antagonist of the P2X3 receptors [87]. It was found to be a close homologue to other spider toxins of unknown function and a more distant homologue to toxins known to bind to other receptors [87]. However, none of the receptors tested, besides P2X3, were sensitive to purotoxin, which makes it to date the only toxin specific of P2X receptors [87].

## 3. Current and Proposed Medical Use of Toxins Targeting the LGIC

Neurotoxins are great sources of medicine or cosmetic products [4]. In addition to their usage as analgesics [79], they are now evaluated as potential treatment for many pathologies [4,6,88]. The toxins presented below are those that are used in clinic or that entered clinical trials (clinical trials were monitored using the website clinicaltrial.org). However, for some LGICs, no toxin has reached clinical trials. Therefore, this section is also complemented with information from patents and recent preclinical data.

An important characteristic of all the toxins presented below is that they are not used as alternative strategies because of toxic properties [89]. Instead, the toxins targeting LGIC are used for their modulating activity.

### 3.1. nAChR

The nAChR has the largest number of known toxins (Sections 2 and 5). It is probably not surprising that nAChR also has the largest number of toxins used medically. Interestingly, both agonists and antagonists are used differently to what is observed for most of the other LGIC.

#### 3.1.1. Agonists

nAChR agonists are considered for their central action and, beyond treatment of tobacco addiction, the main targeted effect is cognition stimulation. 

Three agonists are used to treat tobacco dependence: **Cytisine** (in eastern and central Europe) [90], **Lobeline** [91] and **Nicotine**.

nAChR agonists are also considered in the treatment of neurological disorders. Nicotine is evaluated for Parkinson’s disease (phase II), schizophrenia (phase IV), sarcoidosis (phase IV) and pain (phase IV). Lobeline is evaluated for therapeutic intervention in Attention Deficit Hyperactivity Disorder (phase II). **Epibatidine** has been evaluated for the treatment of pain in phase II clinical trials (as ABT-594) and later abandoned because of adverse effects [4]. **GTS21** (derived from anabaseine) is evaluated (phase II) for therapeutic intervention against schizophrenia [92]. 

#### 3.1.2. Competitive Antagonists

**Curares** derived from d-tubocurarine are widely used for local anesthesia as myo-relaxant. The curare pancuronium is also used for enforcing the death penalty. The reader is referred to the review of Norman Bisset [1] for a historical perspective on the use of curares. 

**Cobratoxin** is used in traditional medicine in China [93] and India. Cobratoxin use has also been proposed for the treatment of small and non-small cell lung cancer by blocking the α7 nAChRs [94,95,96,97,98]. Cobratoxin has also been proposed for the treatment of pain [99] and Multiple sclerosis [100]. In terms of the latter pathology, a chemically attenuated version of cobratoxin [101], also known as receptin or RPI-78M, is under investigation in a phase II clinical trial.

**Conotoxins** hold a significant therapeutic potential that has been reviewed recently [3,4]. α-conotoxin Vc1.1, which targets α9α10 νAChRs, has been tested in a phase II clinical trial (as ACV1) but the development was later discontinued. It should be noted that α-conotoxin Vc1.1 has been proposed to target also the N-type calcium channel [102,103,104]. Muscle-selective α-conotoxins (e.g., α-GI), could represent an alternative to the use of small molecule curare-mimetic muscle relaxants, which are used during surgery, but have slower than ideal recovery period [4,105].

#### 3.1.3. Non-Competitive Antagonists

**Strychnine** used to be prescribed for the treatment of myasthenia until 1930 [106]. “Myasthenia”, however, encompass very diverse pathologies that were not discriminated before the 20th century [107]: (i) *Myasthenia gravis* is an autoimmune disease [107]; (ii) congenital myasthenia syndromes are associated with genetic alterations, some of them increasing or decreasing nAChR response to ACh [108]. Using an antagonist like strychnine cannot be beneficial to treat cases of reduced cholinergic signaling. Such a treatment could only be beneficial for the cases displaying an increased activity of the nAChR, *i.e.*, the slow channel congenital myasthenia. Interestingly, quinidine, which acts as a non competitive antagonist—strychnine has a similar effect on muscle nAChR [109]—is used nowadays to treat patients with slow-channel congenital myasthenia.

**Mecamylamine** is a drug introduced in the 1950s as an anti-hypertensive agent and is now (as TC5214) under clinical trials (phase III) for the treatment of Major Depressive Disorder.

### 3.2. Other Pentameric Ligand Gated Ion Channels

None of the toxins targeting the other pentameric LGIC (GABA, Glycine, Serotonin) have been used in medical practice. Furthermore, none of the toxins targeting these receptors has entered clinical trials. 

For GABA receptors, a patent proposes the use of either the agonist muscimol or the antagonist bicuculine for the treatment of myopia. Numerous, synthetic compounds targeting the GABA receptors are on the market, the most significant being barbiturates and benzodiazepines. These compounds act by increasing the mean open time of the ion channel.

Among synthetic compounds, anesthetics (e.g., propofol) potentiate Glycine receptors. However, propofol also acts on GABA receptors. Currently, no drug targets specifically Glycine receptors [110]. The amino-acid taurine, found in energizing sodas is an inhibitor of the Glycine receptor [111].

Synthetic antagonists targeting the Serotonin receptors are used as antiemitic drugs [112]. Similarly, natural compounds targeting the Serotonin receptors have antiemitic properties, e.g., ginger extracts and delta-9-tetrahydrocannabinol from *Cannabis sativa.*

### 3.3. NMDA Receptors

Toxins targeting the NMDA receptors (Figure 7, top) have neither been used medically nor tested clinically. Synthetic compounds targeting the NMDA receptors in clinical use are mainly channel blockers (Ketamine, Memantine, Amantadine) but also antagonists (Felbamate) [113]. Partial agonists have also been tested clinically (GLYX-13 and Acomposate).

**Conantokins G and T** have been noticed to display antinociceptive [114,115] and anti-epileptic properties [4,116]. Some conantokins demonstrate receptors subunit selectivity, which makes them attractive drug candidates [117].

**Domoic acid** containing algae are used as vermifugal agents in Japanese traditional medicine [118].

### 3.4. AMPA Receptors

Toxins targeting the AMPA receptors (Figure 7, middle) have neither been used medically nor tested clinically. The only drug on the market targeting AMPA receptors is the potentiator Aniracetam (in Italy and Greece). Drugs targeting the AMPA receptors that entered clinical trials were either antagonists (e.g., E2007/perampanel advanced in phase III) or positive allosteric modulators. 

### 3.5. Kainate Receptors

**Kainic acid** is used in Chinese and Japanese traditional medicines as an anthelmintic to treat ascarialis [118]. Kainic acid is the only toxin targeting the kainate receptors (Figure 7, bottom) used as a treatment, and none have been tested clinically.

### 3.6. P2X

**Figure 8:**
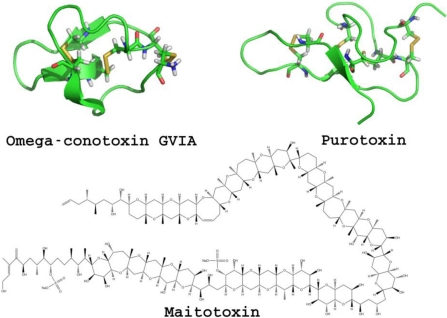
Representative toxins targeting P2X receptors.

No drug targeting P2X receptors is on the market yet [119] and only very few clinical trials have been performed on that target (CE-224535 and GSK-1482160, which are not toxins). This observation can probably be explained by the very recent discovery of this family of LGIC. However, P2X receptors are attracting a lot of interest from pharmaceutical companies as shown by the significant number of patents filled recently [120]. All of the compounds proposed to target P2X receptors are antagonists. In agreement with this observation, among toxins targeting the P2X receptors (Figure 8), **Purotoxin** has been proposed for the treatment of pain based on the observation of antinociceptive activity in animal testing [87].

## 4. Conclusion

One significant observation made during the preparation of this review is that the number of toxins acting on each family of LGIC is highly variable, ranging from a large number acting on nAChR to little acting on others like P2X receptors (see Sections 2 and 5). Two biological explanations can be proposed:

Although LGIC are mainly considered for their role at synapses they are also found in organisms that do not have any synapse [121]. Indeed, homologous proteins have been found in prokaryotes for pentameric [122,123] and tetrameric [124] LGIC but are still to be found for P2X receptors. It can therefore be proposed that P2X receptors appeared more recently in evolution and that the early emergence of pentameric and tetrameric LGIC could have allowed more time for toxin co-evolution.

The physiological roles of the diverse LGIC could also play a role in the imbalance in the number of toxins targeting the LGICs. Indeed, nAChRs, which are largely targeted by toxins, have a prominent role at the neuromuscular junction and, as mentioned above, numerous toxins target that function. Glutamate receptors are also involved in the neuromuscular transduction in invertebrates [125]. In comparison, the role of P2X receptors at the neuromuscular junction is limited in that it does not participate directly in the transmission, but in the neuro-muscular junction development and regeneration [126], and should therefore be a less critical target for prey capture.

In addition to these biological explanations, the asymmetry in the knowledge of toxins targeting LGIC could also be due to the lack of research on the latter identified P2X receptors compared to the first identified nicotinic receptors. Indeed, the targeted receptor is not necessarily obvious from the symptoms and specificity of action may come from other reasons than pharmacological ones, *i.e.*, many toxins can affect receptors present in the CNS whereas they do not reach them physiologically. The known interactions therefore largely depend on specific studies on the effect of toxins on LGIC that are themselves asymmetric.

Given the therapeutic potential of toxins, this observation makes the quest for new toxins targeting P2X receptors an attractive area of research.

## Appendix: List of Toxins Targeting the LGIC

### 1. Nicotinic Acetylcholine Receptors

#### 1.1. Peptides and Proteins

##### 1.1.1. Conotoxins

**α-conotoxins** (Table 1) constitutes the largest group of conotoxins that target muscle nAChRs and/or specific subtypes of neuronal nAChRs in mammals [29]. α-conotoxins can distinguish amongst different subunit arrangements and therefore represent valuable pharmacological tools for research and hold therapeutic promises [127].

**Table 1:** α-conotoxins acting on nAChRs.

Name	*Conus* Specie	Target	References
**GI, GIA, GII**	*geographus*	Muscle nAChR	[27]
**MI**	*magus*	Muscle nAChR	[28]
**SI, SIA and SII**	*striatus*	Selectivity for the distinct interfaces (α/γ or α/δ) of the muscle-type nAChR	[28,29]
**ImI, ImII**	*imperialis*	Selective for α7 nAChR but also effective on α3β4, α3β2	[30]
**BuIA**	*bullatus*	Highest potency for α3- and α6−containing nAChRs	[31]
**CnIA, CnIB **	*consors*	Muscle nAChR	[32]
**Ac1.1a, Ac1.1b**	*achatinus*	Muscle nAChR	[33]
**EI**	*ermineus*	Selective for muscle nAChR, also effective on α3β4, α4β2	[34]
**PnIB, (A10L)-PnIA**	*pennaceus*	Selective for α7, α3β4, α3β2 nAChR	[35,36]
**GIC**	*geographus*	Selective for α3β2	[37]
**MII **	*magnus*	Selective for α3β2, α3β2β3, α6∗ nAChR	[38,39]
**PIA **	*purpurascens*	Selective for α6β2, α6β4, α6α3β2(β3), α6α3β4	[40]
**PIB**	*purpurascens*	Muscle nAChR	[41]
**GID**	*geographus*	α7, α3β2, α4β2	[42,43]
**AuIA, AuIB and AuIC**	*aulicus*	Selectively blocks α3β4 nAChRs	[44]
**EPI **	*episcopatus*	Selective for α7, α3β2, α3β4	[45,46]
**AnIB**	*anemone*	α7, α3β2	[47]
**Vc1.1**	*victoriae*	α9, α3β4, α3(α5)β2	[48,49]
**ArIA, ArIB**	*arenatus*	α7, α3β2, α6α3β2β3	[50]
**PeIA**	*pergrandis*	α9α10,α6α3β2β3,α3β2	[51]
**OmIA**	*omaria*	α7, α3β2	[52]
**TxIA**	* textile*	α3β2	[53]
**Lp1.1**	* leopardus*	α3β2,α6α3β2	[54]
**SrIA, SrIB**	*spurious*	α4β2, muscle type nAChRs	[55]

**α-conotoxins** selectively antagonize the foetal subtype of the mammalian neuromuscular nAChR. A few have been identified: **αA_S_ OIVA and OIVB ***(from C. obscures), ***αA_S_ PeIVA** and **αA_S_ PeIVB** (from *C. pergrandis), ***αA PIVA** (from *C. purpurascens*), **αA EIVA and αA EIVB** (from *C. ermineus), ***αC PrXA** (*C. parius), * or **αS RVIIIA** (*C. radiates).*

**Non-α-conotoxins** act on muscle-type nAChRs. **ψ-conotoxin PIIIE** (from *C. purpurascens*) has a structure similar to that of the voltage-gated Na+ channel-blocking μ-conotoxins and acts as a non-competitive antagonist (perhaps a pore blocker) of the muscle-type nAChR [44]. **ψ-conotoxin PIIIF** has also been identified in *C. purpurascens* and **ψ-PrIIIE in** *C. parius.*

##### 1.1.2. Snake Neurotoxins

**Snake neurotoxins** have the same neuromuscular blocking effects than the plant alkaloid (+)-tubocurarine, but with approximately 15–20-fold greater affinity and poor reversibility of action. They are referred to as curaremimetic neurotoxins or postsynaptic neurotoxins (Table 2). Snake venom α-neurotoxins are polypeptides of 60–74 amino acid residues with 4-5 disulfide bridges, divided as short- (60–62 amino acids, 4 disulphide bridges) and long-chains (66–75 amino acids, 5 disulphide bridges).

**Table 2:** Snake neurotoxins acting on nAChRs.

Name	Source	Target	Structural Group	References
**α-****Bungarotoxin**	*Bungarus multicinctus*	Muscle, α7.	Long-chain α-neurotoxin	[128,129]
**NmmI**	*Naja mossambica mossambica*	Muscle	short-chain α-neurotoxin	[130]
**κ−bungarotoxins**	Bungarus genus	α3β2 and other β2-containing nAChRs.	long-chain neurotoxin	[131]
**α-cobratoxin**	Naja genus (e.g., *Naja kaouthia*)	Muscle, α7	long-chain α-neurotoxins	[132]
**Erabutoxin-a****.**	*Laticauda semifasciata*	Muscle	Short-chain α-toxin	
**erabutoxin-b**	*Laticauda semifasciata*	Muscle	short-chain neurotoxin	[133]
**Toxin-****α**	Naja nigricollis	Muscle	short-chain α-neurotoxin	

The human protein **Lynx1** has been proposed to be evolutionary related to snake venom toxins [134,135]. It binds tightly to nAChRs and inhibits their activation. It has been shown that Lynx1 both decreases nAChR sensitivity to ligands and increases desensitization.

**Denmotoxin** is present in the venom from the Colubrid snake *Boiga dendrophila* (Mangrove Catsnake). It displays remarkable species specificity, being able to interact irreversibly and with high affinity with chick muscle nAChR, but only with low affinity with mouse nAChR [136].

**Waglerins** are polypeptide isolated from the venom of South Asian snake *Tropidolaemus wagleri* consisting of 22–24 amino acids and containing one disulfide bridge. These toxins interact with high affinity with muscle-type nAChR [137].

**Weak neurotoxins** form the group of three fingered toxins consisting of 62-68 amino acid residues with five disulfide bridges characterized by low toxicity. Toxins of this type were later referred to as melanoleuca or miscellaneous-type or non-conventional toxins (Table 3). They bind to neuronal as well as *Torpedo* nAChRs, although with low (micromolar) affinities [138].

Acanthophin is found in death adder venoms; they are rich in a diversity of ‘short-chain’ and ‘long-chain’ postsynaptic neurotoxins that bind to nAChR in skeletal muscle and produce facial and bulbar paralysis.

##### 1.1.3. Natural Toxic Peptides from Other Species

**Philanthotoxin** is found in the venom from the Egyptian digger wasp *Philanthus triangulum* and acts both as competitive and noncompetitive antagonist [148].

**Huwentoxin** is a neurotoxic peptide purified from the venom of the Chinese bird spider *Selenocosmia huwena*. This family consists of several types. Huwentoxin-1 is a lethal neurotoxin that binds to the nAChR and blocks neuromuscular transmission. Huwentoxin-2 blocks neuromuscular transmission and acts cooperatively to potentiate the activity of Huwentoxin-I [149,150].

**Table 3:** Weak neurotoxins acting on nAChR.

Name	Origin	Target	Other specifications	References
**Candoxin**	*Bungarus candidus*	α7 and muscle.		[ 139]
**CM-11, CM-2**	*Naja haje haje* (Egyptian cobra)	Muscle		[ 140]
**CM10, CM12, CM-13b, CM-14**	*Naja haje annulifera*	Neuromuscular Junction		[ 141]
**Cm-9a**	*N. kaouthia*			
**S4C11 **	*N. melanoleucaI*.		neurotoxin Homologue	[ 142]
**S5C1, S5C10**	*Dendroaspis jamesoni kaimosae* (Eastern Jameson's mamba)	Muscle		[ 143]
**S6C4**	*Dendroaspis jamesoni Kaimosae* (jameson’s mamba)		67% sequence identity with Bucandin.	
**γ-bungarotoxin**	*Bungarus multicinctus*	post synaptic action		[ 144]
**WTX **	*Naja kaouthia*	α7 and muscle.		[ 138]
**Wntx-5 **	*Naja sputatrix*	Torpedo α1βγδ, chick α7		[ 145]
**NNA2 **	the Taiwan cobra (N. n. atra)	Muscle	long-neurotoxin homologue	[ 146]
**LSIII**	*Laticauda Semifasciata*	neuromuscular blockade		[ 147]

#### 1.2. Alkaloids

**Cytisine** from the golden chain tree is a natural agonist of nicotinic *α*4*β*2/*α*3*β*4*/*α*7 receptors. Other Cytisine-like analogs are **caulophylline** and **tinctorine** [151].

Comparing Nicotine with cytisine and caulophylline allowed drawing attention on the effect of *pKa* on activity [152]. This observation, in turn, paved the way for the proposal that alkaloids bind through a cation/pi interaction with aromatic residues present in the nAChR orthosteric binding site.

**Anatoxin-a** is a natural agonist produced by diverse cyanobacteria throughout the world [153]. Anatoxin-a is a cyanotoxin considered as an environmental issue as it is found in water bodies and promotes animal deaths [154]. In addition, given that intoxication due to anatoxin-a is possible from drinking water it has been considered that it could be used as biological weapon. For all those reasons numerous detection methods have been developed [155]. Anatoxin-a has nanomolar affinities for α4β2, α3β4* and α7nAChR. An analog is **homoanatoxin**.

**Epibatidine** is a potent nicotinic agonist from Dendrobatid frog (*Epipedobates tricolor*) with antinociceptive properties [156]. Epibatidine has selectivity for α4β2 > α3β4 > α7 > α1β1γδ.

Other nicotinic alkaloids from *Epipedobates tricolor* are ***N*-methylepibatidine**, Epiquinamide and nicotine. **Epiquinamide** is selective for nicotinic receptors containing the β2-subunit.

**Anabasine** is found in various natural sources like plant *Nicotiana glauca*, ant *Aphaenogaster fulva*, marine worm *Paranemertes Peregrina*. Anabaseine is an agoinst for α4β2 and α7 nAChR. **Anabaseine** which is a potent agonist on muscle and neuronal α-Bungarotoxin-sensitive nicotinic receptors [157], is found in tobacco and is also present in small amounts in tobacco smoke. Anabasine has been used as an insecticide.

Histrionicotoxin is a toxin isolated from skin secretions of a Colombian frog, *Dendrobates histrionicus*. It is a potent non-competitive antagonist of nAChR. More than 100 toxins have been identified from the skin secretions of members of the Dendrobatidae family of frogs, especially *Dendrobates* and *Phyllobates*. Members of the genus *Dendrobates* (of which there are at least 44 known species) are also known as "poison dart" or "poison arrow" frogs [158].

**Ferruginine** is a rather weak agonist of neuronal type nAChR from plant *Darlingia ferruginea*, other analogs include **darlingine** [159].

**Carbamylcholine** like acetylcholine activates both nicotinic and muscarinic receptors. The affinity for nicotinic receptors is in the order of α4β2 > α3β4∗ > α7.

**Toxiferines** are isolated from *Strychnos* species. C-toxiferine I (also refered to as toxiferine) is a quaternary alkaloids and, like tubocurarin, is a curare that acts as a competitive antagonist for nicotinic receptors, but is relatively nonselective. They were first found very potent to block the neuromuscular junction [160]. Toxiferine I, among neuronal type nAchR tested was found to bind mainly on α7 subtype [161]. Toxiferines were first used in anesthesia but were abandoned later because their effect was prolonged over too long periods [162].

**Neosurugatoxin**, a complex alkaloid glycoside isolated from a mollusk, *Babylonia japonica* [163], is a potent nicotinic competitive antagonist which blocks both muscle and neuronal nAChR [3,164,165,166].

**Ibogaine** is found principally in the west african *Tabernanthe iboga* were it is used in ceremonies of the *Bwiti* religion [167]. It was used for its anti-addictive properties but was later banned for being addictive [167]. It is a noncompetitive blocker of nicotinic receptors [168], and also interacts with other proteins including NMDA receptors [167].

**Strychnine** is a non-competitive antagonist at α4β2 receptors but acts as a competitive antagonist at α7 receptors [169].

**Coaine** is found in the plant source *Erythroxylon Coca* and is is a noncompetitive blocker in particular for α4β receptors. The quaternary methiodide of cocaine represents another *α*7-selective agonist [170].

**Sparteine,** a lupin alkaloid extracted from *Lupinus luteus*, has been found to be an antagonist for α3β4 receptors. Lupin alkaloids are found in wide variety of plants around the world and are generally toxic [171,172].

Parazoanthoxanthin A is a fluorescent pigment of the group of zoanthoxanthins. It has been shown that Parazoanthoxanthin A demonstrates a dual action on *Torpedo* nAChR; both as a pore blocker and a competitive antagonist [173].

#### 1.3. Others

Lophotoxin from various *Pseudopterogorgia* species, gorgonian soft corals, is a potent and irreversible antagonist for neuromuscular-type nicotine receptors [174,175,176,177]. Lophotoxin appears selective towards α4β2 and α1β1γδ nicotinic receptors [178]. It was found to react with Trp 190 in the α-subunit [179]. Its analogue Bipinnatin B has similar properties [180].

**Luffarins** (K, L and R) as well as **Comentins** (A, B and C) are non-specific antagonists extracted from Australian soft corals [3].

### 2. GABA-A Receptors

**Cicutoxin** from the leaves of *Cicuta virosa* causes convulsion and respiratory paralysis [181].

**Cunaniol** from the leaves of *Clibadium sylvestre* a GABAA antagonist is a potent convulsant [181].

**Ethanol** is apositive modulator of GABA receptors [181].

### 3. Glycine Receptors

**Brucine** is a plant alkaloid found in several species, most notably the Strychnine tree (*Strychnos nux-vomica L.*). **Brucine** is structural related to strychnine.

**Tutin** is a plant toxin found in the tutu plant (genus *Coriaria*). It has powerful convulsant effects.

### 4. Serotonin Receptors

The serotonin receptors have very few known toxins: **Conotoxin GVIIA** (σ-conotoxin; a large 41 amino-acids conotoxin [73]) and **d-tubocurarine** (see nAChRs).

### 5. NMDA

**Ibogaine** see nAChR.

**Ibotenic acid** was first isolated from the fungus *Amanita ibotengutake* [182,183]. The fungus *Amanita muscaria *(fly agaric) got its name from its poisonous actions on flies [184].

**Kaitocephalin** was isolated from the fungus *Eupenicillium shearii* as a glutamate receptor antagonist, which protected from kainate toxicity [185].

Kaitocephalin was latter found to be an antagonist of NMDA and AMPA receptors and also a weak antagonist of the KA-type receptor GluK2 [186].

**Phoneutriatoxin** is isolated from the spider *Phoneutria nigriventer*. It was found to have effect on Glutamate uptake [187,188] and to be specific of NMDA receptors [189].

### 6. AMPA

**DABA** (2,4-diaminobutyric acid) and **BOAA** (beta-*N*-oxalylamino-L-alanine) are found in the seeds of the flat pea *Lathyrus sylvestris*. Both DABA and BOAA are excitatory neurotoxins acting on the AMPA-type receptors [184].

**Philanthotoxins** (alpha, beta, gamma, delta) were first isolated from the wasp *Philanthus triangulum*. δ−philanthotoxin blocks glutamate receptors [190], both AMPA [191] and kainate [192].

Philanthotoxin forms the class of polyamine toxins with argiotoxin, Joro spider toxin and agatoxin. Polyamine toxins are non-competitive antagonist binding probably at the ion channel [193].

**Argiotoxins** were first isolated from the spider *Argiope lobata* [194]. Argiotoxin is an inhibitor of NMDA and kainate receptors [195].

**Joro spider** **toxin** was isolated from the Joro spider *Nephilia clavata* [196]. Joro spider toxin shows analgesic properties [197].

**Willardiine** was first isolated from seeds of *Acacia willardiana * [198] and latter from *Acacia lemmoni, Acacia millefolia, and Mimosa asperata * [198]. Willardiine acts as an agonist on the AMPA and kainate receptors [199].

### 7. Kainate Receptors

Kainic acid is the first membre of the Kainoids familly of molecules that also encompass notably **Domoic acid** and **acromelic acid**. Acromelic acids (A, B and C) are found in the poisonous mushroom *Clitocybe acromelalga* [200]. Domoic acid was originally isolated from the red alga *Chondria armata* and was found in the genus *Pseudonitzschia* and the species *Nitzschia navis-varingica*. Domoic acid is associated with Amnesic shellfish poissoning [85]. Domoic acid also activates AMPA-type receptors [201].

**Dysiserbaine** is a neuroexcitotoxic amino acid isolated from the Micronesian marine sponge *Dysidea herbacea.* Dysiserbaine is another kainate-receptors specific molecule that is structurally unrelated to Kainoids [85].

**Concanavalin A** is a plant lectin isolated from jack bean (*Canavalia ensiformis)* [202]. It was found to be a positive allosteric modulator [203].

### 8. P2X Receptors

**α-Conotoxin GVIA** has first been known to be a N-type calcium channel blocker [204]. It was latter shown to be also a potent inhibitor of P2X3 and P2X2/X3 receptors [205]. The observation that they move dose-response curves to higher ATP concentrations without affecting gating kinetics suggests that it is a non-competitive inhibitor [205].

**α-Hemolysin** promotes the formation of pore leading to hemolysis [206]. As P2X7 are known to open large pores their involvement has been tested. It was shown that α-Hemolysin effect involves P2X1 and P2X7 [206]. It might be speculated that this cytotoxic activity is related to cell death routes involving P2X receptors. In a related manner, *Porphyromonas gingivalis* toxicity requires the secretion of an ATP-consumption enzyme to prevent apoptosis [207].

**Endotoxin**. Lipopolysaccharides have been shown to increase levels of P2X4/P2X7 expression in mouse hypocampus [208]. It was suggested that P2X receptors might have a role in IL-1 release [208] in addition to Toll-like receptor 4 [209]. Indirect effect of LPS could also be through modification of ATP levels [210].

**Maitotoxin** was first proposed to act on P2X7 receptors because pores opened by maitotoxin could not be differenciated from those induced by the agonist Bz-ATP [211]. Thanks to variations in buffers and antagonists it was latter found that the effect is complementary [212].
